# Finite Element Modelling of Bandgap Engineered Graphene FET with the Application in Sensing Methanethiol Biomarker

**DOI:** 10.3390/s21020580

**Published:** 2021-01-15

**Authors:** Paramjot Singh, Parsoua Abedini Sohi, Mojtaba Kahrizi

**Affiliations:** Department of Electrical and Computer Engineering, Concordia University, Montreal, QC H3G1M8, Canada; paramjot.singh@concordia.ca (P.S.); parsoua.sohi@concordia.ca (P.A.S.)

**Keywords:** GFET, methanethiol biosensor, COMSOL modelling, bandgap engineering, DFT, functionalized graphene

## Abstract

In this work, we have designed and simulated a graphene field effect transistor (GFET) with the purpose of developing a sensitive biosensor for methanethiol, a biomarker for bacterial infections. The surface of a graphene layer is functionalized by manipulation of its surface structure and is used as the channel of the GFET. Two methods, doping the crystal structure of graphene and decorating the surface by transition metals (TMs), are utilized to change the electrical properties of the graphene layers to make them suitable as a channel of the GFET. The techniques also change the surface chemistry of the graphene, enhancing its adsorption characteristics and making binding between graphene and biomarker possible. All the physical parameters are calculated for various variants of graphene in the absence and presence of the biomarker using counterpoise energy-corrected density functional theory (DFT). The device was modelled using COMSOL Multiphysics. Our studies show that the sensitivity of the device is affected by structural parameters of the device, the electrical properties of the graphene, and with adsorption of the biomarker. It was found that the devices made of graphene layers decorated with TM show higher sensitivities toward detecting the biomarker compared with those made by doped graphene layers.

## 1. Introduction

Yielding monolayer of honeycomb networked carbon (C) atoms from the graphite leads to one of the most promising materials for photonics and electronics research and industry [1,2]. sp^2^ hybridised carbon in this 2D material, called graphene, reveals remarkable electronic and mechanical properties such as very large mobility of charge carriers and high electric and thermal conductivities [1,2]. Graphene is a zero-bandgap material considered to be a semimetal [3] a the property that conceals its functionalities and limits its applications especially for semiconductor technology [4,5,6]. To overcome this limitation, this hexagonally structured material is transformed into a semiconductor through various band gap engineering methods including creating edge and quantum confinement effects by fabrication of one-dimensional graphene nanoribbons [7,8,9,10,11], passivation with foreign molecules [12,13], as an outcome of straintronics [14,15], and doping with impurities [16,17] and by applying electric field perpendicular to the graphene surface [18,19]. Using the above techniques, not only do we induce a gap in the band structure of the graphene, but we are able to tune the gap. Graphene has potential for many applications, such as graphene field effect transistor (GFET) [20,21,22,23,24], photonic devices such as photodetectors [25,26,27,28,29,30], phototransistors [31,32,33], and mode-locked lasers [34].

Moreover, due to the high surface-to-volume ratio and the possibility of generating high adsorption site density [35], graphene is extensively used in sensor technology. Schedin et. al. [36] developed the first graphene-based gas sensor. The sensor works based on the change in the hall resistance of the graphene layer as the local carrier concentration varies due to the adsorption of NO_2_ molecules on the surface. Thereafter, researchers work extensively on graphene and its derivatives for sensory activities based on piezoelectric effects [37,38,39,40], optical effects [41,42,43], surface phenomenon [44,45,46], and others. The contemporary graphene sensor technology is mostly based on a GFET [20,23,47]. GFETs are reported for sensing several chemical/biomolecules such as OH ions [48], monosodium L-glutamate [49], *Escherichia coli* (*E. coli*) [50], ethanol [51], glucose [52], hydrogen gas [53], exosomes [54], and nitrogen-based gases [55].

In this report, we model, design, and analyse GFET biosensors. For this, graphene is transmuted into semiconducting material using two methods. The first approach is doping graphene with impurities such as boron (B), aluminium (Al), and gallium (Ga) as n-type and nitrogen (N) phosphorous (P) and arsenic (As) as p-type dopants. Decorating the graphene surface with a transition metal (TM) such as palladium (Pd) is the second technique that we use to manipulate the band gap structure of the graphene. Using these techniques not only allows for transferring of the semimetal graphene to a semiconducting material but also gives the capability to generate and control the concentration of adsorption sites on the surface of graphene layers to attach the biomolecules under investigation. The prepared graphene layers are then used to design GFET-based biosensors to detect biomarkers such as methanethiol.

Methanethiol is volatile organo-sulphur material that is considered as a biomarker for the diseases caused by microorganisms like *Helicobacter pylori* bacteria (*H. pylori*) [56], *Porphyromonas gingivalis* (*P. Gingivalis*) *phylum Bacteroidetes* [57,58]. To the best of our knowledge, this is the first time that a graphene-based electronic device is used to detect methanethiol.

Density functional theory (DFT) accompanied by drift-diffusion formalism is used to calculate the physical properties of the semiconducting graphene such as band structure, mobility, and effective density of states. Adsorption energy is also calculated to analyse the surface phenomena on all variants of graphene with or without adsorption of methanethiol. The calculated parameters are used to design and simulate the GFET devices using COMSOL Multiphysics. The dependencies of I-V curves on the electronic properties of the graphene layer as well as the geometrical parameters of the device are considered to evaluate the device’s sensing performance.

## 2. Device Structure

**Figure 1:**
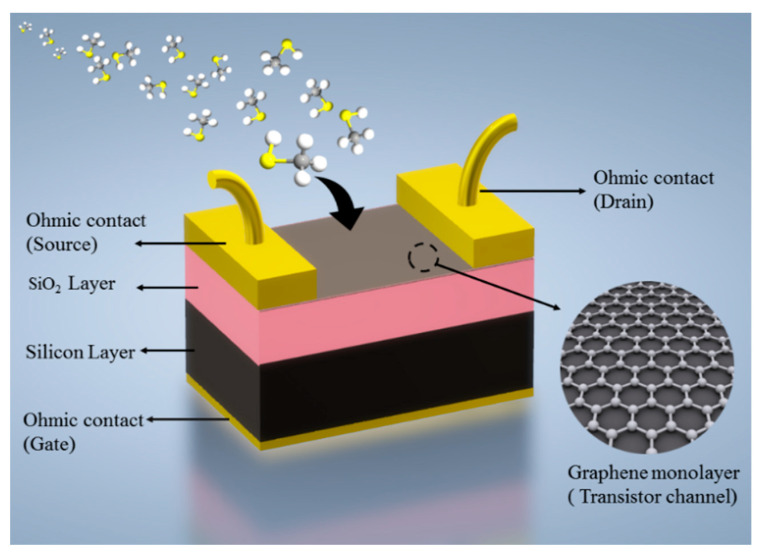
The proposed graphene field effect transistor (GFET) model. The back-gate structure enables binding of the biomarker to the channel surface.

The semiconductor module of COMSOL Multiphysics was used to design and simulate a GFET biosensor. The geometrical configuration of the proposed device is illustrated in Figure 1. The device comprises a one-atom-thick graphene monolayer laying on a silicon-oxide/silicon (SiO_2_/Si) substrate. The SiO_2_ is an insulating layer, serving as a dielectric with a dielectric constant (ɛ_r_) of 3.99. For the sake of calculations, the boundary conditions at all terminals are considered ohmic.

I-V measurements were used to determine the performance and characteristics of the device. These measurements were subjected to varying the dimensions of the graphene channel and its surface chemistry (functionalization), the thickness of the dielectric layer, and the doping concentration of the Si substrate.

The graphene layer serves as a channel in the above structure and must be in the semiconducting state. As it was a semimetal material in nature, we needed to manipulate its band structure to transfer it to a semiconductor. Among several methods to induce bandgap in the graphene band structure, we used the following two methods.

In the first method, the graphene was doped with both donors and acceptors, making it compensated. Here, although graphene is doped with impurities, the material remains intrinsic since the concentration of the introduced n-type and p-type dopants will be the same.

Most common dopants used in graphene are elements from group III for acceptors and from group V for donors (as carbon is in group IV). In this work, the semiconducting properties of graphene are studied for B-N, Al-P, and Ga-As doping.

In the second method, the bandgap opening for graphene arises by adsorption of TMs to the graphene surface. The adsorption happens without introducing crystal defects in graphene lattice. Among TMs, we used Pd due to its high stability and catalytic activities [59,60,61,62,63,64]. The electrical properties of the functionalized graphene layer in the absence and presence of the biomarker are crucial parameters to determine the overall sensing performance of the device. These parameters are evaluated precisely by atomistic analytical studies through DFT implemented in Quantum ATK (QATK) software.

## 3. Results and Discussion

### 3.1. Atomistic Modelling via Quantum ATK

#### 3.1.1. Band Gap Engineering of Graphene

##### Compensated Doping Method

**Figure 2:**
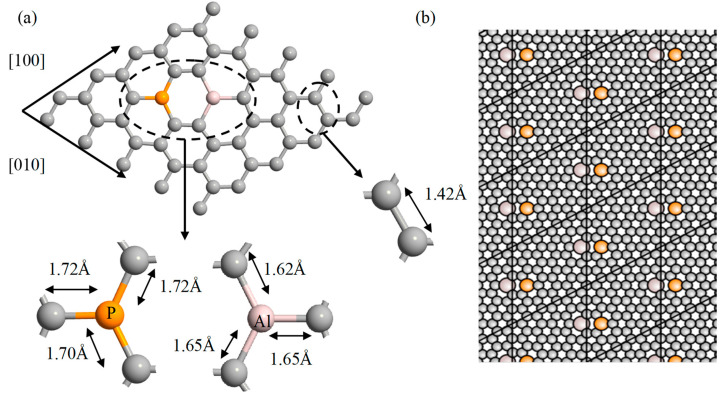
Ground-state optimal structure of Al-P doped graphene; (**a**) optimized bond lengths of Al—C, P—C, and C—C; (**b**) extended structure of co-doped graphene considered for the energy band structure calculations.

Figure 2a illustrates the atomic structure of Al-P doped graphene lattice. Optimization is performed to achieve the lowest energy structure and therefore the most stable atomic conformation. The zoomed-in areas in the figure present the calculated Al—C, P—C and C—C bond lengths of a ground-state optimal structure.

In the proposed structure, the P atom with one extra electron than C atom acts as an n-type impurity by incorporating one electron per nm^2^ into the graphene lattice, whereas the Al atom is one electron deficient and acts as a p-type impurity by incorporating one hole per nm^2^ to the lattice structure.

After geometry optimization, it was found that co-doping of graphene with Al-P atoms did not destroy the Clar’s structure but slightly deformed it, as shown in Figure 2a. Figure 2b illustrates the doped graphene structure with semi-infinite extension in [100] and [010] directions considered for energy-band structure calculations.

The same analyses were also performed for B-N and Ga-As dopants. Each dopant atom was linked with three C atoms and forms different bond lengths. The bond lengths along with the induced bandgap are summarized in Table 1. All the DFT calculations were performed on the same concentration of dopants of one donor–acceptor pair per nm^2^. It was found that the Ga-As pair has induced maximum bandgap as compared to other dopants.

##### Transition Metal Decoration

**Figure 3:**
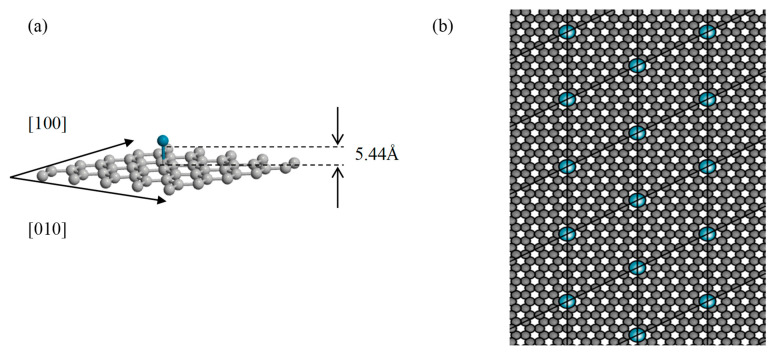
(**a**) The Pd-decorated graphene structure with C—Pd bond length; (**b**) extended surface of Pd decorated graphene considered for the energy band structure calculations.

TMs have high adsorption capabilities compared to other elements [65]. In this method, a Pd hetero atom was used to functionalize graphene surface [66,67,68,69,70,71,72]. The most stable position of a Pd atom on graphene is a bridge position (the position between two consecutive atoms attached through a bond), as shown in Figure 3a [73]. Mulliken population analysis shows that bond formation between C and Pd atom occurs due to interaction between p_z_ orbital of C atom (which is perpendicular to the plane of C atoms) and d orbital of Pd valence band. After optimization, bond length between the Pd and C is 5.44 Å, as depicted in Figure 3a. The extended surface of Pd decorated graphene is shown in Figure 3b with one Pd atom per nm^2^. In semiconductor industries, an appropriate concentration of the dopant is usually considered to eliminate the effects of dopant–dopant interaction. In this work, we considered the 1 Pd atom per/ 1 nm^2^, by which there will be the least interaction between Pd atoms.

#### 3.1.2. Interaction with Methanethiol Biomarker

**Figure 4:**
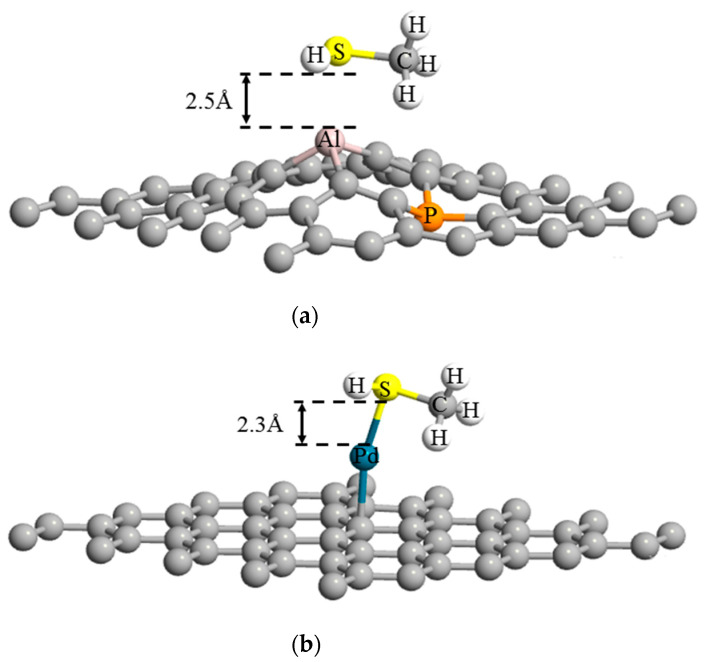
Optimized structures of graphene-biomolecule complex show (**a**) physiosorbed methanethiol biomolecule on Al-P-doped graphene lattice and (**b**) chemisorbed methanethiol biomolecule on Pd-decorated graphene lattice.

Characteristics of the interactions between the methanethiol, (CH_3_SH), and graphene is determined by their binding energy (E_AD_) [74]. Figure 4a,b present the most stable position of methanethiol on the surface of Al-P co-doped and Pd-decorated graphene, respectively, in terms of high adsorption energy.

Compensated doped graphene shows a powerful association with the methanethiol through interaction between sulphur (S) and Al atoms. As illustrated in the Figure 4a, the Al atom relocates itself by rising towards the methanethiol molecule, causing curvature in the structure. However, there is no chemical bond formed between the biomarker and Al-P co-doped graphene, and adsorption of biomarker by the graphene is due to physisorption phenomena. Even though P atoms do not show strong enticement towards the biomarker, it plays a crucial role in opening the bandgap of the graphene. A single impurity atom, like Al or P, opens the Dirac point, but the material remains metallic in nature as some valence bands overlap with Fermi level.

Contrary to what was observed for the case of compensated graphene, Figure 4b illustrates that a bond formation occurs between the methanethiol molecule and Pd-decorated graphene. The involvement of an S atom in bond formation with Pd confirms the occurrence of chemisorption, representing stronger binding between the guest–host complex. Table 2 summarizes the calculated adsorption energies in proposed complexes. The values are counterpoise-corrected interaction energies (E_AD+CP_), which correct the errors arising due to overlapping of basis orbitals of the two interacting atoms [74].

The negative values of the E_AD+CP_ in Table 2 indicates the favourable host–guest molecules adsorption. Furthermore, larger negative E_AD+CP_ corresponds to more efficient adsorption and consequently higher sensing functionalities toward methanethiol. Therefore, the comparison between the values shows that the graphenes doped with B-N and Ga-As do not have efficient adsorption energy towards the indicated biomarker. As a result, for further analyses, we only considered Al-P-doped graphene and Pd-decorated graphene with adsorption energies of −0.90 eV and −1.22 eV, respectively.

##### Electrical Properties of Al-P Doped Graphene before and after Interactions with Methanethiol

**Figure 5:**
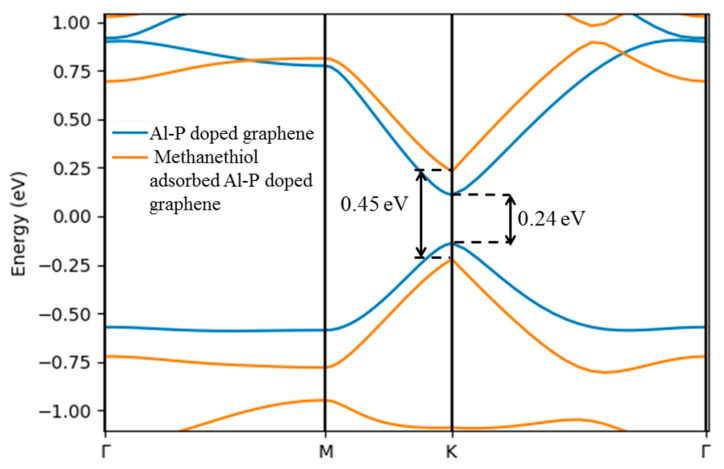
Band structure of Al-P-doped graphene and methanethiol adsorbed Al-P-doped graphene.

Figure 5 illustrates the calculated band structure of Al-P-doped graphene before and after methanethiol adsorption. As is seen, methanethiol adsorption increases the bandgap of the graphene from 0.24 eV to 0.45 eV. However, the intrinsic behaviour of material remains the same after adsorption.

The effective density of states (N_c_ or N_v_) represents the sum of quantum states disseminated on the entire band in the form of two Dirac delta function defined at the edge of the band [75]. N_c_ (N_v_) is a function of the carrier mass ($m_{e}^{\mathrm{dos}}$ or $m_{h}^{\mathrm{dos}}$) [76]. $m_{e}^{\mathrm{dos}}$($m_{h}^{\mathrm{dos}})$ is the longitudinal (transverse) effective mass and depends on the valley degeneracy of the energy bands [77]. In all cases, valley degeneracy is constant, which means only the values of longitudinal and transverse effective masses affect the N_c_ and N_v_. The longitudinal and transverse effective mass of electron decrease as conduction band minima (CBM) and valence band maxima (VBM) converge towards the Fermi level, and they become massless at the Dirac point, which is the same as those of intrinsic graphene. After the adsorption of the biomarker, the bandgap increases, and consequently effective masses increase; as a result, N_c_ and N_v_ increase as tabulated in Table 3. The work function (Φ) expresses the total amount of energy required to knock out one electron from Fermi level to vacuum level [75] and it is inversely proportional to Fermi energy (E_f_). Since adsorption of methanethiol decreases E_f_, it leads to increment in the work function of methanethiol adsorbed complexes. As is summarized in Table 3, Al-P-doped graphene has a work function of 4.2 eV, which increases to 4.5 eV once the biomarker is adsorbed.

Boltzmann Transport Equation (BTE) with relaxation time approximation (RTA) is used to calculate mobility (μ) in graphene structures. μ is directly proportional to the relaxation time of carrier (Ԏ) [78]. Acoustic phonon scatterings have a major contribution to the value of Ԏ at room temperature. In graphene, there are three acoustic phonon modes: longitudinal acoustic (LA), transverse acoustic (TA), and out-of-plane acoustic (ZA) modes [79]. These modes affect the electron–phonon coupling matrix, which is used to calculate Ԏ. As is tabulated in Table 3, our calculations show that carriers mobilities are decreased when methanethiol is adsorbed in Al-P-doped graphene, which affirms that the velocity of the electrons and holes are also decreased. The structural distortion in Al-P graphene during the biomarker adsorption is the major cause of reduction of mobility.

##### Electrical Properties of Pd-Decorated Graphene before and after Interactions with Methanethiol

**Figure 6:**
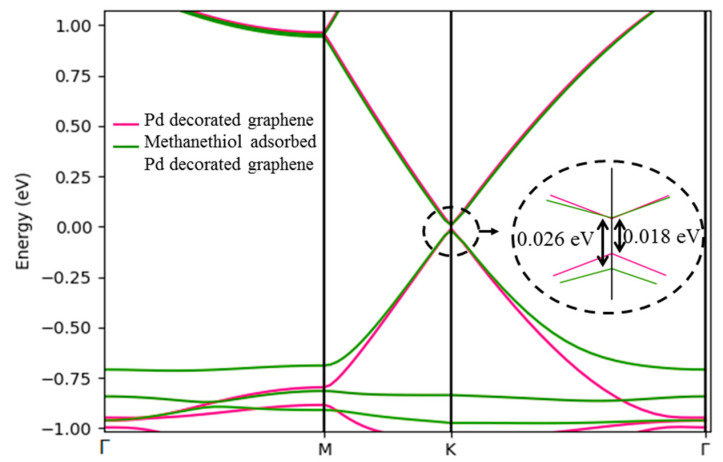
Band structure of Pd-decorated graphene and methanethiol adsorbed Pd-decorated graphene.

The Pd decorated graphene shows n-type behaviour with a bandgap of 0.018 eV. As is illustrated in Figure 6, the adsorption of the biomarker increases the bandgap to 0.026 eV.

Our calculations show that the longitudinal and transverse effective mass of carriers increases as the methanethiol molecule is adsorbed on the graphene. Consequently, N_c_ and N_v_ also increase with adsorption. Biomarker adsorption also reduces the E_F_ of the system as presented in Table 3. Due to the inverse relationship of E_F_ with Φ, Φ of Pd-decorated graphene changes from 4.01 to 4.77 eV after adsorption.

Structural distortion increases the phonon scattering in the lattice, which adversely affects the mobility of the material. Decoration of Pd atom on the graphene decreases the mobility to 10^5^ as it creates distortion in lattice. Pd atom acts as a linking agent between graphene and analyte and impedes graphene distortion. Therefore, adsorption of methanethiol increases mobility as summarized in Table 3.

### 3.2. Device Characterization via COMSOL

COMSOL Multiphysics is used to investigate the DC characteristics of the proposed GFET devices. The software efficiently allows assigning physical properties of materials to model the device. The investigated semiconducting properties of the functionalized graphene in the presence and absence of the biomarker (as summarized in Table 3) are applied in the material properties section required by the model.

The Al-P doped GFET (AlP-GFET) and Pd decorated GFET (Pd-GFET) performances are characterized by considering the effect of geometrical and physical parameters of the devices.

The impact of device parameters variation on the sensitivity of the sensor is discussed in the following sections.

#### 3.2.1. DC Characteristics of Device

**Figure 7:**
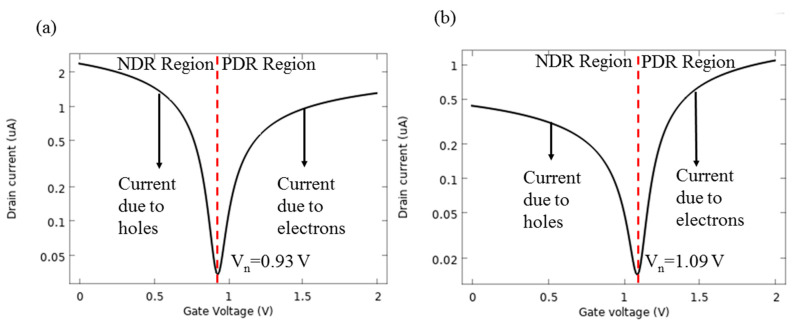
I-V characteristics show two distinct regions and the particular V_n_ for (**a**) AlP-GFET, (**b**) Pd-GFET.

A GFET is a three-electrode device, composed of a graphene channel between two electrodes and a gate contact to modulate the electronic response of the channel. In this section, the electrical characteristics of the AlP-GFET and Pd-GFET devices in the absence of biomarkers are evaluated. For these analyses, the channel length (L, the source-drain spacing) and channel width (W) of the device are considered 2 μm and 1 μm, respectively. A p-type Si with the doping concentration of 10^18^ cm^−3^ is used as the substrate. While the source is grounded, the drain current (I_d_) is measured by sweeping the gate voltage (V_g_) from 0 to 2 V at a drain voltage (V_d_) of 10 mV. I-V characteristics of the devices are shown in Figure 7a (AlP-GFET) and Figure 7b (Pd-GFET). Two distinct regions appear in the graphs: negative differential resistance (NDR) and the positive differential resistance (PDR). A minimum happens as these two regions meet, which is known as the Dirac point at gate voltage, V_n_ (neutral voltage). According to the graphs, the V_n_ of 0.93 V and 1.09 V were observed for AlP-GFET and Pd-GFET, respectively.

This peculiar behaviour of the I-V characteristics of the device is due to the ambipolar transport nature of graphene, which implies the coupled motion of holes and electrons. Therefore, the charge carrier concentration along the graphene channel gives more insight into the I_d_-V_g_ curve discussed in Figure 7.

**Figure 8:**
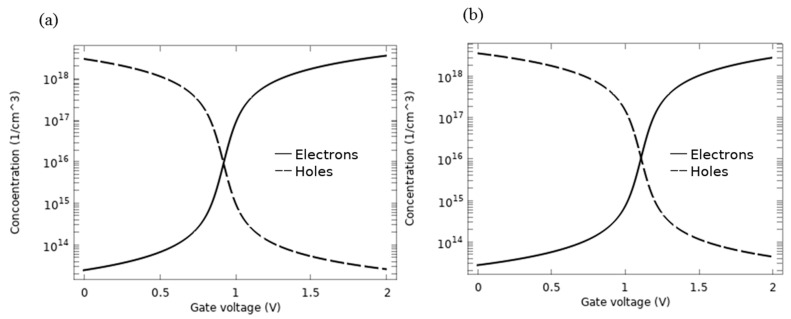
Carrier concentration in the graphene channel versus V_g_ for (**a**) AlP-GFET, (**b**) Pd-GFET. This confirms the occurrence of inversion of the device channel at V_n_.

The density of the carriers for AlP-GFET and Pd-GFET devices versus the gate voltage are shown in Figure 8a,b, respectively. The figure shows that the holes are the majority carrier when the value of V_g_ is below the value of V_n_ of the device. At V_n_, the concentration of both carriers is equal, and as V_g_ increases beyond V_n_, a channel inversion is observed and the electrons will dominate the carrier concentrations.

The conductance of the channel is determined by the abundance of charge carriers, which is influenced by the applied gate voltage. The conductance, in return, determines the I_d_. Therefore, in Figure 7, the negative slope of I_d_ in the NDR region corresponds to the decreasing.

The trend of carrier hole concentration and the positive slope of I_d_ in the PDR region correspond to the increasing trend of the carrier electron concentration.

**Figure 9:**
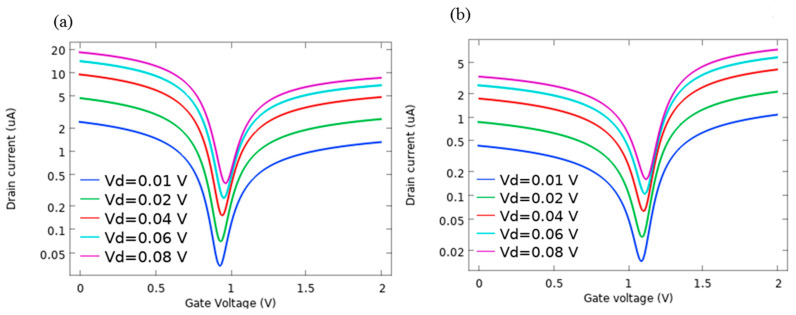
I-V characteristics as a function of applied V_d_ for (**a**) Al-P doped GFET (**b**), Pd-decorated GFET. According to the graphs, V_n_ of the devices remains constant as V_d_ varies.

Figure 9a illustrates the I_d_-V_g_ curve of AlP-GFET at different drain voltages. It is observed that with an increment of the drain voltage, the I-V curve shifts toward the higher current values. The V_n_ of 0.93 V remains nearly constant for all V_d_ values. I_d_-V_g_ characteristics of Pd-GFET shown in Figure 9b follow the same trend as discussed for AlP-GFET. For this device, V_n_ is at the voltage of 1.09 V, which is higher than what is observed for AlP-GFET. Moreover, the lower current intensity of Pd-GFET is due to the lower carrier mobilities in this device compared to AlP-GFET, as summarized in Table 3.

**Figure 10:**
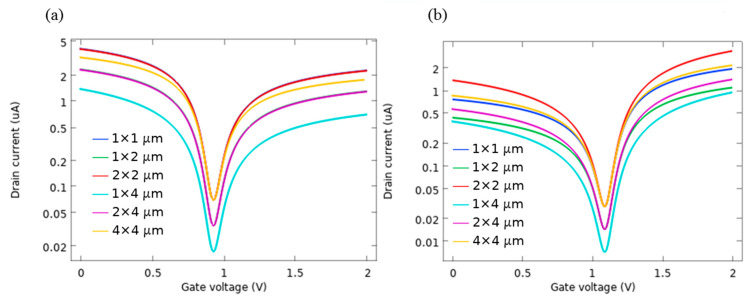
I-V characteristics as a function of channel dimension (W × L), for (**a**) AlP-GFET (**b**) Pd-GFET. The I_d_ of the devices with equal $\frac{W}{L}$ ratios are overlapped.

To give a more authenticated perception to the device study, I_d_-V_g_ curves were calculated at V_d_ of 10 mV for the devices with different channel dimensions (W × L). Figure 10a presents the obtained results for the AlP-GFET devices, and Figure 10b presents those for Pd-GFET devices. As the results show, the value of V_n_ for both devices remains constant and is independent of the device geometry. This is one of the positive characteristics of the device that shows the sensing properties of the device will not be affected by the structural device dimensions. However, the device I_d_ is a function of channel length and width, $I_{d}\propto \frac{W}{L}$. The effect of the channel dimension on the device I_d_ follows the following equation [80].

(1) $$I_{d}=\frac{W}{L}\;\int_{V_{s}}^{V_{d}}{(q\mu }_{p}{p+\;q\mu }_{n}n+q\frac{\mu_{p}{+\mu }_{n}}{2}n_{\mathrm{pud}})$$

where the parameters W and L are width and length of the channel, respectively; $\mu_{n}$ and $\mu_{p}$ are electron and hole mobility, respectively; and $\sigma \;={q\mu }_{n}n+{q\mu }_{p}p$ is the conductivity of the channel. The term, $q\frac{\mu_{p}+\mu_{n}}{2}n_{\mathrm{pud}}$ denotes that the residual charge occurs due to the spatial homogeneity.

**Figure 11:**
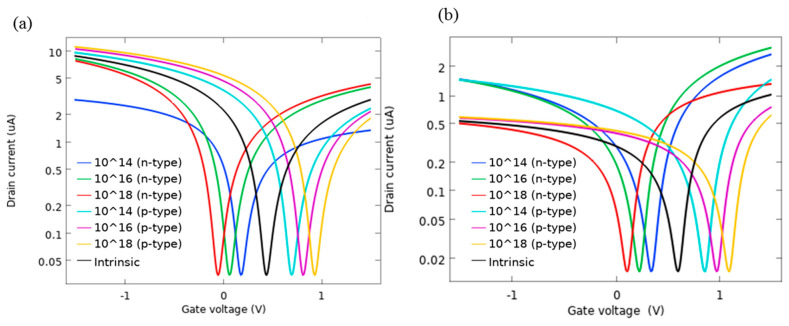
I-V characteristics as a function of doping type and concentration for (**a**) AlP-GFET, (**b**) Pd- GFET.

We also investigated the characteristics of the device with respect to electrical properties of the silicon substrate for both variants of the device. The results show that they have a significant effect on the value of V_n_ as we change the doping type and its concentration of the substrate. The I-V characteristics in Figure 11a (AlP-GFET) and Figure 11b (Pd-GFET) illustrate that as the concentration of dopants increases, the position of V_n_ moves toward higher voltages for p-type substrates, while it moves toward lower voltages for the n-type substrate case.

**Figure 12:**
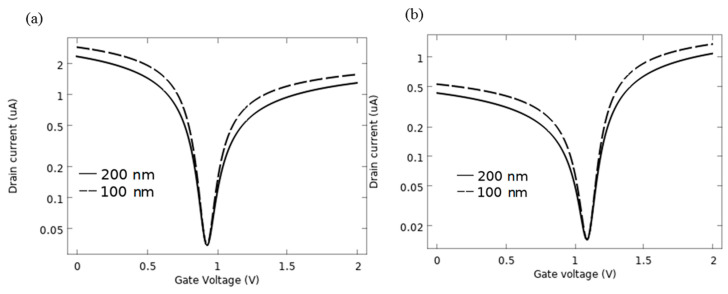
I-V characteristics as a function of dielectric layer thickness (**a**) AlP-GFET (**b**) Pd-GFET.

Figure 12a shows the measured I_d_-V_g_ for the AlP-GFET device, and Figure 12b shows it for the Pd-GFET device for two different oxide thicknesses, 200 nm and 400 nm. According to the graphs, the variation of the oxide layer thickness does not affect the sensing properties of the devices as the value of V_n_ remains the same for both cases.

#### 3.2.2. Detection of Methanethiol Biomarker

To check the sensitivity of the device against biomarkers, the device DC characteristics are typically evaluated in the presence of a biomarker and compared with a reference test in the absence of biomarkers.

For this study, a device with channel dimensions of 1 × 2 μm^2^ (W × L) and a p-type substrate with doping concentration 10^18^ cm^−3^ is considered. V_g_ is swept from 0 to 2 V, while a constant V_d_ of 10 mV is applied on the drain. One biomolecule per nm^2^ was considered for the adsorption concentration of the biomarker on the graphene layer.

**Figure 13:**
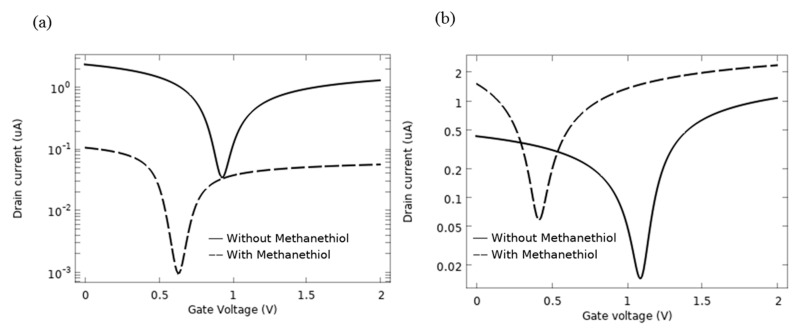
I-V characteristics before and after adsorption of methanethiol for (**a**) AlP-GFET, (**b**) Pd-GFET.

Figure 13a illustrates the I_d_-V_g_ characteristics of the AlP-GFET device, and Figure 13b illustrates it for the Pd-GFET device, in the presence of the methanethiol as compared to a reference test in absence of the biomarker.

I_d_-V_g_ curves obtained for AlP-GFET show a clear shift in V_n_ from 0.93 V to 0.63 V after the adsorption of methanethiol by the channel surface, while for the Pd-GFET, it changed from 1.09 V to 0.42 V. The shift in V_n_ is attributed to the change of μ, bandgap, N_c_ or N_v_, and Φ of the functionalized graphene once it is exposed to the methanethiol molecule. As summarized in Table 3, these physical quantities show variation after adsorption of methanethiol, which affects the V_n_ value. The observed shift in V_n_ for the Pd-GFET is larger than that of the AlP-GFET, which affirms the higher sensitivity to the biomarker for this device.

It is observed that the overall current intensity for AlP-GFET decreases after the adsorption of the biomarker. However, for the case of Pd-GFET, the intensity of the current slightly increases after biomarker adsorption. This behaviour of the device corresponds to the effect of adsorption on the carrier mobility of the graphene channel (as summarized in Table 3). The increase in mobility of the carriers will be correlated with an increase in I_d_ of the device and vice versa.

Based on the results discussed in Section 3.2.1, the V_n_ of the device is only a function of semiconductive properties of the graphene channel and varies by functionalization of the graphene and its interaction with the molecules. The geometry of the device and the biasing parameters does not alter the V_n_, and therefore the sensitivity of the sensors towards exposure to methanethiol could be simply defined as $S≅\left|\frac{\Delta \mathrm{Vn}}{\mathrm{Vn}}\right|$. The calculated sensitivity of the AlP-GFET towards methanethiol when the V_n_ changes from 0.93 V to 0.63 V is 32.25%; similarly, V_n_ in the Pd-GFET changes from 1.09 V to 0.42 V, which shows 60.55% sensitivity towards this biomarker.

There are other reports in the literature regarding applying methanethiol as a biomarker to develop biosensors [81,82,83]. Compared to those, the present work proposes a design to develop a device with higher sensitivity and simpler structure.

## 4. Conclusions

In this work, we demonstrated the possibility of using a GFET biosensor for the detection of methanethiol biomarker, which is used to identify diseases caused by microorganisms such as *Helicobacter pylori* bacteria (*H. pylori*) and *Porphyromonas gingivalis* (*P. Gingivalis*) *phylum Bacteroidetes*. Surface chemistry of the graphene layers is manipulated using two methods: (a) doping the graphene crystals and (b) decorating the surface of the graphene with TMs before using it as the channel of the GFET. The device was designed and characterized using COMSOL Multiphysics. The NDR characteristic of the graphene, which is due to its ambipolar behaviour, was observed using three-terminal measurements. The deep that appeared in the I-V characteristics of the device is used to study the sensitivity of the sensors. The results show that the device characteristic is only sensitive to the doping level of the silicon substrate; otherwise, it is independent of other physical parameters of the device. However, our investigations show that electrical properties of the functionalized graphene layers particularly in the absence and presence of the biomarkers are largely affected the characteristics of the sensor. The device is more sensitive to the adsorbed biomarker in the case of Pd-GFET compared to the devices developed using Al-P doping.

## Figures and Tables

**Table 1:** Induced bandgap and C—dopant bond length in different doped graphene.

Material		B-N Doped Graphene	Al-P Doped Graphene	Ga-As Doped Graphene
Bandgap (eV)		0.24	0.24	0.55
Bond length 1 (Å)	Acceptor	1.47	1.62	1.71
	Donor	1.4	1.7	1.7
Bond length 2 (Å)	Acceptor	1.47	1.65	1.72
	Donor	1.41	1.72	1.72
Bond length 3 (Å)	Acceptor	1.48	1.65	1.72
	Donor	1.41	1.72	1.72

**Table 2:** The total energies, adsorption energies between biomarker and graphene.

Material	E_AD+CP_ (eV)
Methanethiol adsorbed Pd decorated graphene	−1.22
Methanethiol adsorbed B-N doped graphene	−0.01
Methanethiol adsorbed Al-P doped graphene	−0.90
Methanethiol adsorbed Ga-As doped graphene	−0.02

**Table 3:** Physical parameters of different variants of graphene before and after methanethiol adsorption calculated by DFT analysis.

	Al-P Doped Graphene	Methanethiol Adsorbed Al-P Doped Graphene	Pd Decorated Graphene	Methanethiol Adsorbed Pd Decorated Graphene
$E_{g}$ (eV)	0.24	0.45	0.018	0.026
$E_{\mathrm{VBM}}-E_{F}$ (eV)	0.12	0.23	0.011	0.019
$E_{\mathrm{CBM}}-E_{F}$ (eV)	0.12	0.23	0.007	0.007
$E_{F}$ (eV)	5.32	4.99	4.97	4.88
Nature	Semiconductor	Semiconductor	Semiconductor	Semiconductor
$\frac{m_{l\left(e\right)}}{m_{e}}$	0.048	0.160	0.003	0.004
$\frac{m_{t1\left(e\right)}}{m_{e}}$	0.099	1.909	0.007	0.010
$\frac{m_{t2\left(e\right)}}{m_{e}}$	0.092	0.174	0.005	0.008
$\frac{m_{l\left(h\right)}}{m_{e}}$	0.052	0.164	0.003	0.004
$\frac{m_{t1\left(h\right)}}{m_{e}}$	0.108	2.287	0.007	0.010
$\frac{m_{t1\left(h\right)}}{m_{e}}$	0.098	0.176	0.005	0.008
$\frac{m_{e}^{\mathrm{dos}}}{m_{e}}$	0.120	0.596	0.00748	0.011
$\frac{m_{h}^{\mathrm{dos}}}{m_{e}}$	0.130	0.641	0.00748	0.011
$N_{c}$(cm^−3^)	1.04 × 10^18^	1.15 × 10^19^	1.61 × 10^16^	2.88 × 10^16^
$N_{v}$(cm^−3^)	1.17 × 10^18^	1.28 × 10^19^	1.61 × 10^16^	2.88 × 10^16^
Φ(eV)	4.200	4.500	4.010	4.700
χ (eV)	4.080	4.270	4.003	4.693
$\mu_{e}$ (cm^2^/Vs)	1.25 × 10^5^	1.79 × 10^4^	9.58 × 10^4^	1.28 × 10^5^
$\mu_{h}$(cm^2^/Vs)	2.29 × 10^5^	4.00 × 10^4^	3.5 × 10^4^	1.90 × 10^5^

## Data Availability

The data that support the findings of this study are available from the corresponding author upon reasonable request.
